# A Case of Aconite Poisoning

**Published:** 1869-01-15

**Authors:** John W. Henley

**Affiliations:** Yates City, Knox Co., Ills.


### *A Case of Aconite Poisoning.*

BY JOHN W. HENLEY, M. D., YATES CITY, KNOX CO., ILLS.

*Editor Chicago Medical Journal:*

Aconite is becoming so extensively used by the medical profession, both as an internal and external remedy ; and being a powerful acro-narcotic poison, for which there is known no direct antidote, the following may interest many readers of THE JOURNAL.

Dec. 31st ult., was called in great haste to visit Mrs. Varney, five miles from town. She had been confined two days previous, and was directed to take one tablespoonful of castor oil on third day after accouchement. I had sent by her husband the following mixture, for a neighbor woman, to be used as a liniment for neuralgia of the face:

R. Tinc. Aconit, rad. f ℥ jss:

Chloroformi.

Spts. Rectificat, aa f ℥ j;

Oil Oliv. ℥ j;

Misce.

Of which one table-spoon was administered to Mrs. V. for castor oil. I arrived at the scene of confusion in about two hours and a half after the poison had been swallowed. Found that she had been induced to swallow two large tablespoonfuls of castor oil immediately after the mistake was discovered. She was suffering no acute pain, and what is a little strange, there was no gastric iritation, had been no vomiting, nor the slightest nausea. There was considerable depression of nervous energy, a pricking and benumbing sensation in mouth, throat and limbs, with a sense of fullness all over the body. The most disagreeable sensations she complained of, were a coldness about the stomach, and the unnatural and large feeling of the limbs. The pulse were 80 and rather full, respiration free and easy. The surface of the body was but very little colder than natural. The sense of taste was entirely wanting. After a hasty survey of the condition of the case, I gave two teaspoonfuls of ground mustard in warm water, with a view of producing speedy emesis. She neither tasted the mustard, which was fresh ground, nor felt the slightest effect of it in the stomach. After waiting some 15 minutes I gave nearly one drachm ipecac, in two doses, following with copious draughts of warm water, sweetened. In a short time she vomited freely, which was followed by free evacuations of the bowels. All the time the above emetics were being administered the limbs were rubbed with stimulating lotions, which afforded much relief, as the friction destroyed in part the disagreeable tingling and sense of fulness. The patient now began to feel a great sense of depression. The pulse were weak and down to 48. Extremities cold. In short, there seemed to be a great tendency to death by asthenia. Gave whisky sling, with ginger, freely and repeatedly. In one hour the patient was bathed in a copious perspiration, and began to revive. In two hours more I left her safe. The only inconvenience she experienced afterward was the profuse perspiration and consequent prostration, which lasted about forty-eight hours.

The dose of strong fine aconite root was undoubtedly a fatal one if it had been administered alone. And what was there in the combination which served at least as a partial antidote? It could not have been the alcohol, for there was not a sufficient quantity to even afford temporary safety. Neither could it have been the olive oil, for there was scarcely a half drachm in the quantity given. But the olive oil contained in the mixture, and the castor oil given immediately after the poison was swallowed no doubt served in part to mitigate the acrid properties of the aconite. And there is no doubt in my mind but the chloroform was the agent in the mixture which saved the patient until other means were applied to eliminate the poison. For the effects of the aconite and chloroform being opposite, the one tends to counteract the effects of the other. And since my experience with this case I feel confident that the best remedies for poisoning from aconite, are, aside from evacuating the stomach by active emetics or stomach pumps, oils to counteract the acrid properties of the drug, and powerful stimulants to prevent the depression of nervous energy and brain. Among the latter let me suggest as paramount, chloroform, with the yolk of an egg, and water, to be given according to the amount of depression.

The suggestions here made, and facts gained in the treatment of poisoning by aconite may with equal propriety prove efficient in destroying the dangerous effects of an over-dose of the Gelsiminum Sempervirens. And I would suggest to Dr. Hani, of Indiana, who reports a case of fatal poisoning by this drug, in Dec. 1st number of THE JOURNAL, that he waste not valuable time in administering opium and strychnia as antidotes to this poison, but use more direct stimulants as soon as practicable, and thereby sustain the vital powers until the narcotic has spent its force.

